# Beneficial Effects of L-Carnitine Supplementation during IVM of Canine Oocytes on Their Nuclear Maturation and Development In Vitro

**DOI:** 10.3390/ani11020581

**Published:** 2021-02-23

**Authors:** Adel R. Moawad, Ali Salama, Magdy R. Badr, Mohamed Fathi

**Affiliations:** 1 Department of Theriogenology, Faculty of Veterinary Medicine, Cairo University, Giza 12211, Egypt; ash_andro@yahoo.com (A.S.); mido2022@yahoo.com (M.F.); 2 Department of AI and ET, Animal Reproduction Research Institute, Agriculture Research Centre, Giza 12556, Egypt; magdybadr69@yahoo.com

**Keywords:** oocyte, dog, IVM/IVF, L-Carnitine, embryo

## Abstract

**Simple Summary:**

In vitro production of canine embryos is a technique that can be used as a model to conserve endangered species and to establish efficient breeding systems for domestic dogs. However, compared with other species, the success rates of in vitro embryo production (IVEP) in dogs are low. L-Carnitine (LC) is a small water-soluble molecule; it plays an essential role in fatty acid metabolism and acts as a potent antioxidant. Various studies have reported the beneficial impacts of LC on IVEP in many mammalian species other than dogs. Therefore, these experiments investigated the effects of LC supplementation during in vitro maturation (IVM) on canine oocytes maturation, fertilization, and development in vitro. We show that the supplementation of IVM media with LC has positive impacts on oocyte maturation, fertilization, and preimplantation embryo development rates. We also demonstrate that 0.6 mg/mL LC is the most beneficial concentration to be used. It resulted in significantly higher maturation, fertilization, and embryo developmental rates than the control and other LC concentrations. These outcomes are essential for refining the IVM conditions that can advance the efficiency of assisted reproductive technologies (ARTs) in dogs.

**Abstract:**

This study aimed to investigate the effect of L-Carnitine (LC) supplementation during in vitro maturation (IVM) of canine oocytes on nuclear maturation, fertilization status, and preimplantation development. Cumulus–oocyte complexes (COCs) collected from the ovaries of ovariohysterectomized female dogs were matured in vitro for 72 h in a TCM-199 medium supplemented with (0.1, 0.3, 0.6, 1.0, or 2.0 mg/mL) or without (0.0 mg/mL) LC. Matured oocytes were fertilized in vitro with frozen–thawed spermatozoa, and zygotes were cultured in a SOF medium for 7 days. IVM rates were higher (*p ≤* 0.05) in 0.3 and 0.6 mg/mL LC supplemented groups than in the control (0.0 mg/mL LC) and other LC groups. Fertilization (18 h postinsemination (pi)) and cleavage (2–16-cell stage at day 3 pi) rates were higher (*p ≤* 0.05) in the 0.6 mg/mL LC group than in the control and 0.1, 1.0, and 2 mg/mL LC supplemented groups. Interestingly, 4.5% of fertilized oocytes developed to morula (day 5 pi) in the 0.6 mg/mL LC group, which was higher (*p ≤* 0.05) than those developed in the 0.3 mg/mL group (1.0%). No cleaved embryos developed to morula in other groups. In conclusion, LC supplementation at 0.6 mg/mL during IVM of canine oocytes improved their maturation, fertilization, and preimplantation embryo development rates following IVF and in vitro culture (IVC).

## 1. Introduction

Assisted reproductive technologies (ARTs) are required in dog breeding programs to enhance domestic dog reproduction and serve as a model for the conservation of wild and endangered canid species. However, the development of ARTs, for example, in vitro maturation of oocytes (IVM), in vitro fertilization (IVF), and embryo culture, has lagged in dogs compared to other domestic species [1]. Failure to develop ARTs in canids results from peculiar species-specific reproductive characteristics of dogs [1]. Dogs are a nonseasonal and monoestrous species, and they ovulate only once or twice a year at an interval of 5–12 months [2]. Canid oocytes also ovulate at an immature stage compared with other mammalian species (pre-germinal vesicle breakdown) and require 48–72 h in the oviduct postovulation to complete nuclear maturation [2]. The canine oocyte’s maturation rate is low compared with other domestic species, and only up to 20–40% reach the metaphase II (MII) stage [3,4]. Various factors have been reported that impact the meiotic maturation and in vitro development of canid oocytes, including donor age, stage of the reproductive cycle, size of ovarian follicles, culture media, and culture duration [5,6]. It has been reported that the low IVM rates in dogs could be because of inadequate culture conditions that hinder oocytes’ ability to resume meiosis [7]. Low IVM rates in dogs are also associated with defects in fertilization and embryo development rates [3]. For example, very few morulae and blastocysts developed following IVF of in vitro matured dog oocytes [8,9,10], yet no puppies were born from these attempts. Even when in vivo matured oocytes were used, the outcomes were low, and only 7 puppies were born out of 19 transferred embryos [11]. It has recently been reported that IVF of in vivo matured oocytes begets in 66.67% of two-cell embryo development (18 embryos/27 oocytes) [12]. However, injection of CRISPR-Cas9 into in vivo matured oocytes before IVF (pre-IVF) or in one-cell zygotes after IVF (post-IVF) resulted in lower cleavage rates (43.1% and 38.8%, respectively) [12]. Two healthy puppies were born following a transfer of 15 (pre-IVF) and 13 (post-IVF) embryos [12]. However, none of the control embryos produced live births [12]. Previous studies speculated that IVM of dog oocytes using conventional media and culture conditions is challenging, mainly due to the high intracellular lipids, namely lipid yolk, within the oocytes [6,13]. Analysis of total lipids extracted from canine oocytes has revealed that intracellular lipids are composed of saturated fatty acids, triglycerides, cholesterol, phospholipids, and glycolipids [6,14]. Furthermore, dogs differ from other domestic species in that the oocyte metabolically consumes a high level of glucose via glycolysis [15]. Additionally, the oocyte’s developmental ability to achieve nuclear maturation is tightly associated with the metabolism of glucose and glutamine [6,15]. Despite multiple publications, standard systems for in vitro production (IVP) of canine embryos have not yet been developed, and new approaches are needed [16].

L-Carnitine (LC) is a small water-soluble molecule present in most tissues and body fluids. It participates in the mitochondria by beta-oxidation [17]. It also has an antioxidant activity that protects the cells against oxidative stress and DNA damage [18,19]. Positive effects of LC on IVEP have been previously reported in many mammalian species. In pigs, LC improved oocyte maturation and embryo development rates, which were linked with an enhancement in mitochondrial activity and reduced intracellular lipid contents and H_2_O_2_ levels [20,21]. In mice, supplementation of the IVM medium with LC improved spindle configuration and chromosome alignment in metaphase II (MII) oocytes, and it enhanced embryonic development through a decrease in apoptosis [22,23]. In camels, LC improved nuclear maturation, fertilization, and blastocyst rates after IVM/IVF and embryo culture [24]. In cattle, LC promoted relocation of active mitochondria to the inner oocyte membrane and subsequently enhanced embryonic development [25]. However, there has been no analysis of the importance of LC concerning canine oocytes’ metabolism and development. The present study aimed to investigate the effect of LC supplementation during IVM of canine oocytes on their nuclear maturation, fertilization status, and preimplantation development following IVF and embryo culture.

## 2. Materials and Methods

All chemicals and reagents were purchased from Sigma–Aldrich (St. Louis, MO, USA) unless stated otherwise.

### 2.1. Ovary Collection and Recovery of Cumulus–Oocyte Complexes (COCs)

Ovaries were obtained from different breeds of healthy domestic bitches at random stages of the estrous cycle (*n* = 54, 1 to 6 years old) via routine ovariohysterectomy in the local Egyptian Society for Mercy to Animals (ESMA) under the supervision and approval of the shelter’s owners. Ovaries were transported to the laboratory within 1–2 h in a thermos flask containing sterile phosphate-buffered saline at 37 °C. After transportation, the fat and ligaments were trimmed off carefully and discarded. COCs were released by repeatedly slicing the ovarian cortex with a surgical scalpel blade at room temperature. COCs were then placed in 35-mm Petri dishes containing HEPES-buffered TCM-199 (H-TCM 199) and examined under a stereomicroscope. After three washes in the same medium, COCs with homogeneous dark cytoplasm and three or more layers of compact cumulus cells were chosen for IVM [1,4].

### 2.2. IVM and the Evaluation of Nuclear Maturation

For IVM, groups of 5 to 10 selected COCs were cultured in vitro in 100-μL droplets of the maturation medium, namely TCM-199 with Earle’s salts, supplemented with 5 μg/mL oFSH, 5 μg/mL oLH, 10% FCS, 50 μg/mL sodium pyruvate, and 50 μg/mL gentamycin for 72 h at 39 °C in 5% CO_2_ in the air (control group) [4,26]. The IVM medium was covered under mineral oil and pre-equilibrated in the same conditions for at least 4 h before the start of IVM. For LC-treated groups, COCs were matured under the same conditions in an IVM medium supplemented with 0.1, 0.3, 0.6, 1.0, or 2.0 mg/mL LC. The LC doses were selected based on previous studies in other species [22,24]. Nuclear maturation was assessed in denuded oocytes (cumulus cells were removed by gentle pipetting in a hyaluronidase-containing medium) using 1% orcein staining according to the method previously described [4]. Stained oocytes were examined under a phase-contrast microscope. Based on the chromatin configuration, oocytes with MII plates were considered mature.

### 2.3. IVF and Evaluation of Fertilization Status

For IVF, four frozen semen straws obtained from two mature male dogs were thawed in a water bath at 37 °C for 30 s [4]. Thawed semen samples were pooled and resuspended in a 2 mL sperm-TALP medium supplemented with 5 mM caffeine and then centrifuged at 500× *g* for 5 min. Following centrifugation, the supernatant was discarded, and the pellet was resuspended in 1 mL sperm-TALP. Sperm suspension was kept for 30 min at 39 °C in 5% CO_2_ in the air for the swim-up. Groups of 10 matured COCs were inseminated with motile spermatozoa at 2 × 10^6^ sperm cells/mL in 100 µL of fertilization-TALP medium supplemented with 6 mg/mL BSA, 50 μg/mL gentamycin, and 5 mM caffeine [4]. Gametes were coincubated together for 18 h at 39 °C in 5% CO_2_ in the air. Eighteen hours postinsemination (pi), the signs of fertilization were determined by staining the denuded oocytes with 1% orcein staining. Stained oocytes were examined under a phase-contrast microscope. They were categorized as fertilized when their ooplasm contained a swollen sperm head or a male and female pronuclei [4].

### 2.4. In Vitro Culture and Embryo Evaluation

After 18 h pi, cumulus cells and attached spermatozoa were removed from inseminated oocytes by gentle pipetting in a hyaluronidase-containing medium. Groups of five presumptive zygotes were then cultured in 50-μL drops of an embryo culture medium (synthetic oviductal fluid; SOF supplemented with 1 mg/mL BSA) under mineral oil at 39 °C in a humidified atmosphere of 5% CO_2_, 5% O_2_, and 90% N_2_ until day 7 (day 0 = day of insemination). The culture medium was changed every 48 h [27]. Preimplantation embryo development was evaluated under a stereomicroscope, and the proportions of cleaved embryos (2–16 cells), morula, and blastocyst development were assessed on days 3, 5, and 7 pi, respectively [4].

### 2.5. Statistical Analysis

Three replicates were applied for each experimental group. Data were presented as percentages and analyzed by the chi-square test. Statistical analysis was conducted by GraphPad Prism 5 software. The results were considered to be statistically significant at *p ≤* 0.05.

## 3. Results

### 3.1. Effects of LC Supplementation on Nuclear Maturation Rates

As shown in Table 1, the proportions of mature oocytes in 0.3 and 0.6 and mg/mL LC supplemented groups were higher (*p ≤* 0.05) than in the control (0.0 mg/mL LC) and other experimental groups.

### 3.2. Effects of LC Supplementation on Fertilization Events

As shown in Table 2, the highest fertilization rate was achieved in the 0.6 mg/mL LC supplemented group (25.9%). This value was higher (*p ≤* 0.05) than in the control group (0.0 mg/mL LC) and 0.1, 1.0, and 2.0 mg/mL LC supplemented groups (values ranged from 12.2% to 14.6%). No significant differences were observed between 0.6 and 0.3 mg/mL LC groups. Supplementation of the IVM medium with 0.3 mg/mL LC significantly increased (*p ≤* 0.05) fertilization rates in comparison to those of the group supplemented with 2.0 mg/mL (21.4% vs. 12.2%).

### 3.3. Effects of LC Supplementation on Preimplantation Embryo Development

Supplementation of the IVM medium with 0.6 mg/mL LC significantly increased (*p ≤* 0.05) the rate of 2–16-cell embryo development on day 3 pi compared to the control group and the 0.1, 1.0, and 2.0 mg/mL LC supplemented groups. No significant differences were observed in the 2–16-cell embryo development percentage between 0.6 and 0.3 mg/mL LC groups (Table 3). Interestingly, 4.5% of IVM/IVF oocytes developed to morula (day 5 pi) in a 0.6 mg/mL LC supplemented group. This value was higher (*p ≤* 0.05) than that produced in the 0.3 mg/mL group (1.0%). A similar trend was also observed when we calculated the percentage of morula development based on the number of cleaved embryos (the values were 27.3% vs. 7.7% in 0.6 and 0.3 mg/mL LC, respectively). No embryos developed beyond the 2–16-cell stage in the rest of the groups (Table 3).

## 4. Discussion

In vitro production of canine embryos is a technique that can be used as a model to conserve endangered species and to establish efficient breeding systems for domestic dogs. However, compared with other species, the success rates of IVM/IVF and in vitro embryo development are low in canids. Therefore, the present study aimed to improve the efficiency of in vitro maturation and canine oocytes’ development. L-Carnitine acts as an essential cofactor for beta-oxidation; it regulates ATP production from lipids and is a potent antioxidant [28]. We demonstrated that LC supplementation during IVM improved maturation, fertilization, and the development of canine oocytes. We also showed that 0.6 mg/mL LC is the most beneficial concentration to be used. It resulted in higher maturation and embryo developmental rates than the control and other LC concentrations.

The decisive role of LC during IVM and embryo culture has been previously reported in many species, including mice [18,22,23,29,30], pigs [20,21,31], cattle [32], and sheep [33,34]. The beneficial effect of LC is likely attributed to its antioxidant capacity and its ability to increase ATP production from intracellular lipid stores [22,23,28,29,30]. The antioxidant effects of L-Carnitine have been confirmed in vitro in somatic cells [35,36] and oocytes [37]. During IVM, oocytes may experience oxidative damage resulting in mitochondria and DNA perturbations and subsequent impairment in fertilization and embryonic development [22,23,38,39]. Canine oocytes require up to 72 h IVM duration to achieve a high nuclear maturation rate [4,9]. During this long culture period, oocytes may experience a damaging effect on the endogenous antioxidant systems, resulting in ROS accumulation that is detrimental to oocytes’ developmental competence [5,6,15]. Besides, dog oocytes contain many intracellular lipids and are likely to be susceptible to oxidative stress [6]. Therefore, we can speculate from the current study that supplementation of LC at 0.3 and 0.6 mg/mL during IVM is crucial in protecting dog oocytes against oxidative stress. Several studies have demonstrated the link between the inclusion of L-Carnitine during IVM and the reduction in intracellular ROS and the increase in the antioxidant enzymes within the oocyte of many species, including mice [37], cattle [40], pigs [21,31], and sheep [34]. The nuclear maturation rates reported here in 0.3 and 0.6 mg/mL LC supplemented groups (35.4% to 41.4%) were higher than those reported previously for dog oocytes cultured in vitro for 48 h (11.9% to 18.6% [27]; 2.2% to 13.3% [41]; 8.4% to 14.7% [7]; 25.6% [9]; 27.3% [42]; and 11.7% to 17.7% for oocytes collected from small (<1 mm) and medium (1–2 mm) follicles, respectively [15]). However, in the study of Songsasen et al. [15], the collection of oocytes from large (>2 mm) follicles resulted in comparable maturation rates (37.7%) to our 0.3 and 0.6 mg/mL LC groups. The maturation rate reported in the 0.6 mg/mL LC supplemented group was also higher than those demonstrated previously when dog oocytes cultured in vitro for 72 h (0.0% to 6.82% [1], 25.5% [4], 2.48% to 13.23% [43], 5.56% to 21.82% [44], and 3.8% [45]). Our findings suggest that LC could positively impact oocyte maturation during a prolonged culture period [25,46]. Regarding the effect of LC on fertilization status following IVF, our results show that treatment of oocytes with 0.6 mg/mL LC during IVM improved fertilization rates compared with the control group and other LC concentrations. These findings indicate that LC exerts a beneficial effect not only during IVM but also postfertilization. Higher fertilization rates observed in 0.6 mg/mL LC treated groups may be due to the higher proportions of matured oocytes noticed in this concentration. The fertilization rate reported here in the 0.6 mg/mL LC group (25.8%) was higher than reported previously in dogs (15.8%, 12/76; 19%, 10/52) [4,9], assuring the positive impacts of LC on IVF of canine oocytes. Previous studies also reported lower pronucleus formation rates (1.85% to 7.69%) in IVM dog oocytes fertilized by fresh or cooled dog sperm [47]. The beneficial effects of LC supplementation during IVM on IVF rates have also been reported in different domestic species [24].

The beneficial effect of LC exists not only during IVM and IVF but also in the preimplantation embryo development of canine oocytes. Our current findings demonstrate that supplementation of IVM media with 0.6 mg/mL LC improved the percentages of cleaved embryos (2–16-cell stage) and morula development at days 3 and 5 pi, respectively (Table 3). In the meantime, we saw only morula development in 0.3 and 0.6 mg/mL LC groups (1.0% and 4.5%, respectively). These results indicate that the inclusion of 0.6 mg/mL LC during IVM of dog oocytes could support embryo development during IVC. Attempts at IVF of IVM dog oocytes previously resulted in low embryo development rates and no live births [8,9,12,24,26,27,47]. For example, only 2% of inseminated dog IVM oocytes developed to eight-cell embryos at 72 h post-IVF [42]. Otoi et al. [8] reported that only one canine oocyte developed to the blastocyst stage following IVM/IVF and IVC. Another study revealed that the IVM of dog oocytes for 48 h resulted in 2.9% 16-cell embryos and 1% morula development after IVF and embryo culture; however, none of the oocytes matured for 72 h developed to these stages [9]. The morula percentage reported in [9] is lower than that reported here in a 0.6 mg/mL LC supplemented group (4.5%). It is known that culturing oocytes for a long period increases ROS accumulation and reduces the antioxidant defense mechanism within the oocytes, creating oxidative stress that harmfully impacts oocyte developmental potential [25,46]. This notion could explain the low rates of embryo development reported in the oocytes matured for 72 h in the study of Otoi et al. [9]. High rates of embryo development seen in the current research in the 0.6 mg/mL LC group indicate that L-Carnitine prevented the harmful consequence of long maturation time. Recent studies showed that LC could prevent oocyte aging and enhance embryos’ developmental capacity from aged oocytes by reducing oxidative stress [25,46]. Previous studies reported that 2.76% (7/253) of in vitro matured dog oocytes could develop to 2–5-cell embryos after IVF, from which only two embryos advanced to the 6–8-cell stage within the 7-day IVC period [47]. Another study demonstrated that 1.4% to 4.1% of IVM/IVF dog oocytes could develop to the 8–16-cell stage at 96–120 h pi without further progress beyond this stage [27].

Substantial research shows that the IVM culture media composition affects COC metabolism and metabolic rate, and the developmental competence of the oocytes is affected by their metabolism [48]. Besides, the processes of oocyte growth, maturation, fertilization, and subsequent preimplantation embryo development require the generation of a sufficient amount of ATP [49]. The utilization of carbohydrates, glucose, pyruvate, and lactate for ATP production has been well identified in the COC [48]. However, fatty acid metabolism via beta-oxidation is a potent energy source with an energy production capacity several-fold higher than that of carbohydrates [28]. Production of energy from intracellular stores of the oocyte during IVM is crucial in species with high levels of stored lipids, such as dogs [6,28]. Beta-oxidation of fatty acids requires the essential cofactor L-Carnitine to transport activated fatty acids from the cytosol into the mitochondria. The addition of L-Carnitine to in vitro cell line cultures is known to increase beta-oxidation [28,50]. The beneficial impacts of L-Carnitine on dog oocyte development reported in the current study could be due to its implications for the energy production of intracellular lipid stores within the oocyte. The link between L-Carnitine and beta-oxidation of fatty acids and the metabolism of canine oocytes warrants further investigation.

## 5. Conclusions

In summary, L-Carnitine supplementation during IVM of canine oocytes improved their nuclear maturation, fertilization rates, and subsequent preimplantation embryo development following IVF and embryo culture. Among the different concentrations used, 0.6 mg/mL LC seems to be the most beneficial one as it produced higher rates of day 5 morula development. These outcomes are crucial for the refinement of IVM conditions that can advance the efficiency of in vitro embryo production in dogs.

## Figures and Tables

**Table 1:** Effects of L-Carnitine supplementation during in vitro maturation (IVM) on nuclear maturation rates of canine oocytes (*n* = 3).

L-Carnitine (mg/mL)	Number of Examined Oocytes	Maturation Rate, *n* (%)
0	197	46 (23.4) ^a^
0.1	216	54 (25.0) ^a^
0.3	209	74 (35.4) ^b^
0.6	227	94 (41.4) ^b^
1	206	49 (23.7) ^a^
2	203	41 (20.1) ^a^

Values with different superscripts (^a,b^) in the same column are significantly different (*p ≤* 0.05).

**Table 2:** Effects of L-Carnitine supplementation during IVM on fertilization rates of canine oocytes following in vitro fertilization (IVF) (*n* = 3).

L-Carnitine (mg/mL)	Number of Examined Oocytes	Fertilization Rate, *n* (%)
0	171	24 (14.0) ^ac^
0.1	192	28 (14.6) ^ac^
0.3	192	41 (21.4) ^ab^
0.6	205	53 (25.9) ^b^
1	195	28 (14.4) ^ac^
2	181	22 (12.2) ^c^

Values with different superscripts (^a,b,c^) in the same column are significantly different (*p ≤* 0.05).

**Table 3:** Effects of L-Carnitine supplementation during IVM on preimplantation embryo development of canine oocytes following IVF and IVC (*n* = 3).

L-Carnitine (mg/mL)	Number of Oocytes	2–16-Cell Stage *n* (%)	Morula/Oocytes, *n* (%)	Total Number of Cleaved Embryos/Oocyte, *n* (%)	Morula/Cleaved Embryos (%)
0	171	11 (6.4) ^a^	0 (0.0) ^a^	11 (6.4) ^a^	0 ^a^
0.1	196	15 (7.7) ^ab^	0 (0.0) ^a^	15 (7.7) ^a^	0 ^a^
0.3	195	26 (13.3) ^bc^	2 (1.0) ^a^	28 (14.4) ^b^	7.7 ^a^
0.6	202	33 (16.3) ^c^	9 (4.5) ^b^	42 (20.8) ^b^	27.3 ^b^
1	184	14 (7.6) ^ab^	0 (0.0) ^a^	14 (7.6) ^a^	0 ^a^
2	187	13 (6.9) ^a^	0 (0.0) ^a^	13 (6.9) ^a^	0 ^a^

Values with different superscripts (^a,b,c^) in the same column are significantly different (*p ≤* 0.05).

## Data Availability

All data sets obtained and analyzed during the experiment are available on fair request from the respective author.
